# Tetra­aqua­bis[1,1′-(4-methoxy­naph­thalene-1,3-diyldimethyl­ene)pyridinium-3-carboxyl­ate-κ*O*]cobalt(II) bis­(perchlorate) hexa­hydrate

**DOI:** 10.1107/S1600536808013123

**Published:** 2008-05-14

**Authors:** Guo-Hua Wang, Feng-Bo Xu, Qing-Shan Li

**Affiliations:** a State key laboratory of Elomento-Organic Chemistry, Nankai University, Tianjin 30071, People’s Republic of China

## Abstract

In the molecule of the centrosymmetric title compound, [Co(C_25_H_20_N_2_O_5_)_2_(H_2_O)_4_](ClO_4_)_2_·6H_2_O, the Co atom is octa­hedrally coordinated by four water mol­ecules lying in the equatorial plane and two monodentate carboxyl­ate groups from two dicarboxylate ligands. The crystal structure involves O—H⋯O and O—H⋯Cl hydrogen bonds..

## Related literature

For related literature, see: Li *et al.* (2006 ▶).
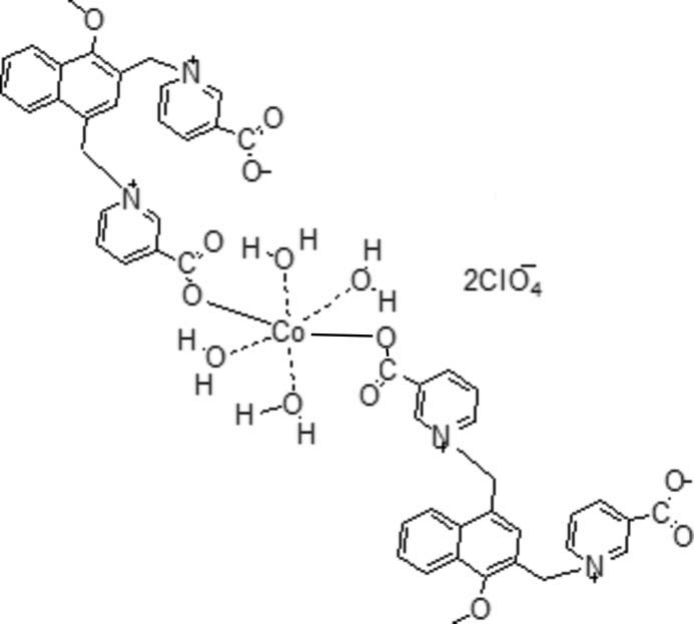


## Experimental

### 

#### Crystal data

[Co(C_25_H_20_N_2_O_5_)_2_(H_2_O)_4_](ClO_4_)_2_·6H_2_O
*M* *_r_* = 1294.86
Triclinic, 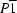

*a* = 7.9162 (19) Å
*b* = 12.703 (3) Å
*c* = 14.757 (3) Å
α = 71.159 (6)°
β = 89.759 (8)°
γ = 77.175 (7)°
*V* = 1365.7 (5) Å^3^
*Z* = 1
Mo *K*α radiation
μ = 0.51 mm^−1^
*T* = 113 (2) K
0.18 × 0.16 × 0.14 mm

#### Data collection

Rigaku Saturn diffractometer
Absorption correction: multi-scan (Jacobson, 1998 ▶) *T*_min_ = 0.914, *T*_max_ = 0.932
12559 measured reflections
4738 independent reflections
4261 reflections with *I* > 2σ(*I*)
*R*_int_ = 0.024

#### Refinement

*R*[*F* ^2^ > 2σ(*F* ^2^)] = 0.057
*wR*(*F* ^2^) = 0.128
*S* = 1.08
4738 reflections
417 parameters
17 restraints
H atoms treated by a mixture of independent and constrained refinement
Δρ_max_ = 1.27 e Å^−3^
Δρ_min_ = −0.45 e Å^−3^

### 

Data collection: *CrystalClear* (Rigaku, 2002 ▶); cell refinement: *CrystalClear*; data reduction: *CrystalClear*; program(s) used to solve structure: *SHELXS97* (Sheldrick, 2008 ▶); program(s) used to refine structure: *SHELXL97* (Sheldrick, 2008 ▶); molecular graphics: *CrystalStructure* (Rigaku, 2002 ▶); software used to prepare material for publication: *CrystalStructure*.

## Supplementary Material

Crystal structure: contains datablocks I, global. DOI: 10.1107/S1600536808013123/gw2035sup1.cif

Structure factors: contains datablocks I. DOI: 10.1107/S1600536808013123/gw2035Isup2.hkl

Additional supplementary materials:  crystallographic information; 3D view; checkCIF report

## Figures and Tables

**Table 1:** Hydrogen-bond geometry (Å, °)

*D*—H⋯*A*	*D*—H	H⋯*A*	*D*⋯*A*	*D*—H⋯*A*
O10—H10*A*⋯O13^i^	0.84 (4)	1.83 (4)	2.665 (5)	171 (6)
O10—H10*B*⋯O1^ii^	0.85 (4)	1.84 (4)	2.668 (6)	166 (6)
O11—H11*A*⋯O12^i^	0.87 (4)	1.83 (4)	2.690 (6)	174 (7)
O11—H11*B*⋯O14^iii^	0.86 (4)	1.85 (4)	2.701 (5)	173 (6)
O12—H12*A*⋯O1	0.83 (4)	1.84 (5)	2.654 (6)	164 (8)
O12—H12*B*⋯O4	0.87 (4)	1.91 (4)	2.772 (6)	173 (8)
O13—H13*A*⋯O5^iv^	0.86 (4)	1.94 (4)	2.787 (6)	172 (7)
O13—H13*B*⋯O9^v^	0.86 (4)	1.95 (4)	2.815 (6)	175 (7)
O13—H13*B*⋯Cl1^v^	0.86 (4)	2.69 (6)	3.451 (4)	147 (6)
O14—H14*A*⋯O4^i^	0.86 (4)	1.88 (4)	2.724 (6)	165 (7)
O14—H14*B*⋯O5^vi^	0.86 (4)	1.98 (4)	2.811 (6)	160 (7)

Symmetry codes: (i) 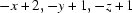; (ii) 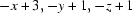; (iii) 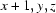; (iv) 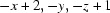; (v) 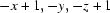; (vi) 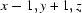.

**Fig. 1.**
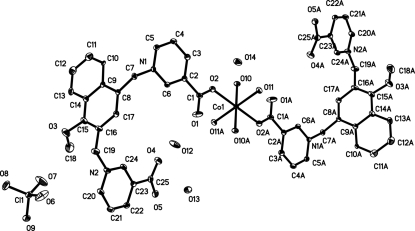
The molecular structure of (I), shown with 30% probability displacement ellipsoids.

## supplementary crystallographic information

### Comment

Recently, corrdination chemistry becomes more and more important in the structural design of supramolecular chemistry. The deprotonated carboxyl group, which easily coordinated to metal atoms and can be used to prepare soluble metal complexes. During the synthesis of polymeric complexes using 3-methoxyl-1,3-pyridinium-3-carboxylate (*L*) as bridging ligand, to our surprise, the title monomeric Co complex was obtained.

As shown in Fig. 1, the stucture of the title compound, (I), four water molecules and two monodentate carboxylate groups from *L* ligands corrdinate to Co. the other three water molecules and carboxylate group of the ligand *L* and the perchlorate anion are free from corrdination.

For related literature, see: Li *et al.* (2006).

### Experimental

An aqueous and water (V/V=1:1) solution of *L* (0.042 g, 0.1 mmol)and Co(ClO4)6H2O (0.11 g, 0.3 mmol) was stirred at 333 K for 10 min and then left to stand at room temperature. Single crystals of (I) were obtained after 3 d.

### Refinement

(type here to add refinement details)

### Crystal data

**Table:**

[Co(C_25_H_20_N_2_O_5_)_2_(H_2_O)_4_](ClO_4_)_2_·6H_2_O	*Z* = 1
*M**_r_* = 1294.86	*F*_000_ = 673
Triclinic, *P*1	*D*_x_ = 1.574 Mg m^−^^3^
*a* = 7.9162 (19) Å	Mo *K*α radiation λ = 0.71070 Å
*b* = 12.703 (3) Å	Cell parameters from 4046 reflections
*c* = 14.757 (3) Å	θ = 2.6–27.8º
α = 71.159 (6)º	µ = 0.51 mm^−^^1^
β = 89.759 (8)º	*T* = 113 (2) K
γ = 77.175 (7)º	Prism, colorless
*V* = 1365.7 (5) Å^3^	0.18 × 0.16 × 0.14 mm

### Data collection

**Table:**

Rigaku Saturn diffractometer	4738 independent reflections
Radiation source: fine-focus sealed tube	4261 reflections with *I* > 2σ(*I*)
Monochromator: graphite	*R*_int_ = 0.024
Detector resolution: 7.31 pixels mm^-1^	θ_max_ = 25.0º
*T* = 113(2) K	θ_min_ = 2.7º
ω scans	*h* = −9→9
Absorption correction: multi-scan(Jacobson, 1998)	*k* = −13→15
*T*_min_ = 0.914, *T*_max_ = 0.932	*l* = −17→17
12559 measured reflections	

### Refinement

**Table:**

Refinement on *F*^2^	Secondary atom site location: difference Fourier map
Least-squares matrix: full	Hydrogen site location: inferred from neighbouring sites
*R*[*F*^2^ > 2σ(*F*^2^)] = 0.057	H atoms treated by a mixture of independent and constrained refinement
*wR*(*F*^2^) = 0.128	*w* = 1/[σ^2^(*F*_o_^2^) + (0.0385*P*)^2^ + 4.0784*P*] where *P* = (*F*_o_^2^ + 2*F*_c_^2^)/3
*S* = 1.08	(Δ/σ)_max_ = 0.004
4738 reflections	Δρ_max_ = 1.27 e Å^−^^3^
417 parameters	Δρ_min_ = −0.45 e Å^−^^3^
17 restraints	Extinction correction: SHELXL97 (Sheldrick, 2008), Fc^*^=kFc[1+0.001xFc^2^λ^3^/sin(2θ)]^-1/4^
Primary atom site location: structure-invariant direct methods	Extinction coefficient: 0.013 (3)

### Special details

**Table:**

Geometry. All e.s.d.'s (except the e.s.d. in the dihedral angle between two l.s. planes) are estimated using the full covariance matrix. The cell e.s.d.'s are taken into account individually in the estimation of e.s.d.'s in distances, angles and torsion angles; correlations between e.s.d.'s in cell parameters are only used when they are defined by crystal symmetry. An approximate (isotropic) treatment of cell e.s.d.'s is used for estimating e.s.d.'s involving l.s. planes.
Refinement. Refinement of *F*^2^ against ALL reflections. The weighted *R*-factor *wR* and goodness of fit *S* are based on *F*^2^, conventional *R*-factors *R* are based on *F*, with *F* set to zero for negative *F*^2^. The threshold expression of *F*^2^ > σ(*F*^2^) is used only for calculating *R*-factors(gt) *etc*. and is not relevant to the choice of reflections for refinement. *R*-factors based on *F*^2^ are statistically about twice as large as those based on *F*, and *R*- factors based on ALL data will be even larger.

### Fractional atomic coordinates and isotropic or equivalent isotropic displacement parameters (Å^2^)

**Table:**

	*x*	*y*	*z*	*U*_iso_*/*U*_eq_	
Co1	1.5000	0.5000	0.5000	0.0166 (3)	
N1	0.7742 (5)	0.6740 (4)	0.2020 (3)	0.0175 (9)	
N2	0.7187 (6)	0.1380 (4)	0.2728 (3)	0.0228 (10)	
O1	1.1365 (7)	0.4585 (4)	0.4208 (5)	0.073 (2)	
O2	1.2985 (5)	0.5855 (3)	0.3906 (3)	0.0245 (9)	
O3	0.6931 (6)	0.2768 (4)	0.0159 (4)	0.0443 (12)	
O4	1.1873 (5)	0.1697 (3)	0.3646 (3)	0.0325 (10)	
O5	1.2889 (5)	−0.0172 (3)	0.4167 (3)	0.0321 (10)	
C1	1.1687 (7)	0.5520 (5)	0.3763 (4)	0.0277 (13)	
C2	1.0353 (7)	0.6348 (5)	0.2975 (4)	0.0220 (12)	
C3	1.0486 (7)	0.7466 (5)	0.2532 (4)	0.0268 (13)	
H3	1.1446	0.7717	0.2701	0.032*	
C4	0.9214 (7)	0.8213 (5)	0.1842 (4)	0.0266 (13)	
H4	0.9292	0.8982	0.1538	0.032*	
C5	0.7833 (7)	0.7839 (4)	0.1596 (4)	0.0217 (11)	
H5	0.6946	0.8352	0.1130	0.026*	
C6	0.8963 (7)	0.6001 (4)	0.2702 (4)	0.0203 (11)	
H6	0.8862	0.5235	0.2997	0.024*	
C7	0.6205 (7)	0.6347 (5)	0.1771 (4)	0.0222 (12)	
H7A	0.5414	0.7000	0.1288	0.027*	
H7B	0.5567	0.6114	0.2354	0.027*	
C8	0.6653 (7)	0.5364 (4)	0.1382 (4)	0.0213 (12)	
C9	0.7197 (7)	0.5518 (5)	0.0436 (4)	0.0238 (12)	
C10	0.7556 (11)	0.6557 (5)	−0.0161 (4)	0.0432 (19)	
H10	0.7389	0.7190	0.0066	0.052*	
C11	0.8159 (8)	0.6675 (6)	−0.1092 (5)	0.0381 (15)	
H11	0.8491	0.7356	−0.1454	0.046*	
C12	0.8263 (8)	0.5787 (6)	−0.1474 (5)	0.0387 (15)	
H12	0.8620	0.5871	−0.2104	0.046*	
C13	0.7823 (8)	0.4757 (5)	−0.0902 (4)	0.0307 (14)	
H13	0.7852	0.4160	−0.1161	0.037*	
C14	0.7340 (7)	0.4612 (5)	0.0052 (4)	0.0243 (12)	
C15	0.6947 (7)	0.3564 (5)	0.0618 (5)	0.0260 (13)	
C16	0.6448 (7)	0.3420 (4)	0.1539 (5)	0.0278 (14)	
C17	0.6314 (7)	0.4329 (5)	0.1918 (4)	0.0243 (12)	
H17	0.5982	0.4223	0.2555	0.029*	
C18	0.8347 (13)	0.1867 (7)	0.0329 (7)	0.068 (3)	
H18A	0.9385	0.2157	0.0128	0.082*	
H18B	0.8166	0.1373	−0.0035	0.082*	
H18C	0.8504	0.1427	0.1016	0.082*	
C19	0.5813 (7)	0.2382 (5)	0.2098 (5)	0.0327 (15)	
H19A	0.5253	0.2121	0.1638	0.039*	
H19B	0.4911	0.2610	0.2508	0.039*	
C20	0.6727 (7)	0.0366 (5)	0.3076 (4)	0.0261 (12)	
H20	0.5597	0.0311	0.2914	0.031*	
C21	0.7884 (8)	−0.0581 (5)	0.3660 (4)	0.0280 (13)	
H21	0.7556	−0.1290	0.3907	0.034*	
C22	0.9537 (7)	−0.0498 (5)	0.3889 (4)	0.0249 (12)	
H22	1.0363	−0.1157	0.4272	0.030*	
C23	0.9980 (7)	0.0553 (4)	0.3556 (4)	0.0209 (11)	
C24	0.8756 (7)	0.1483 (4)	0.2974 (4)	0.0223 (12)	
H24	0.9033	0.2210	0.2744	0.027*	
C25	1.1779 (7)	0.0706 (5)	0.3813 (4)	0.0241 (12)	
Cl1	0.30440 (18)	0.00621 (11)	0.11433 (10)	0.0254 (4)	
O6	0.2137 (9)	0.0363 (5)	0.1891 (3)	0.072 (2)	
O7	0.4485 (8)	0.0568 (5)	0.0958 (6)	0.075 (2)	
O8	0.1939 (7)	0.0440 (4)	0.0298 (3)	0.0463 (13)	
O9	0.3657 (6)	−0.1168 (3)	0.1445 (3)	0.0324 (10)	
O10	1.6526 (5)	0.6076 (3)	0.4204 (3)	0.0222 (8)	
H10A	1.604 (7)	0.678 (4)	0.399 (4)	0.033*	
H10B	1.724 (7)	0.597 (5)	0.467 (4)	0.033*	
O11	1.4046 (5)	0.6184 (3)	0.5691 (3)	0.0241 (9)	
H11A	1.293 (5)	0.645 (5)	0.563 (5)	0.036*	
H11B	1.447 (7)	0.676 (5)	0.566 (5)	0.036*	
O12	0.9425 (6)	0.3085 (4)	0.4369 (4)	0.0470 (14)	
H12A	0.985 (10)	0.365 (5)	0.431 (6)	0.070*	
H12B	1.017 (9)	0.260 (5)	0.418 (6)	0.070*	
O13	0.5220 (6)	0.1760 (3)	0.6610 (3)	0.0319 (10)	
H13A	0.589 (8)	0.128 (5)	0.639 (4)	0.048*	
H13B	0.562 (9)	0.160 (6)	0.720 (3)	0.048*	
O14	0.5114 (6)	0.8080 (3)	0.5631 (4)	0.0372 (11)	
H14A	0.598 (7)	0.828 (6)	0.582 (5)	0.056*	
H14B	0.444 (8)	0.871 (5)	0.528 (5)	0.056*	

### Atomic displacement parameters (Å^2^)

**Table:**

	*U*^11^	*U*^22^	*U*^33^	*U*^12^	*U*^13^	*U*^23^
Co1	0.0134 (5)	0.0155 (5)	0.0214 (6)	−0.0038 (4)	−0.0016 (4)	−0.0065 (4)
N1	0.018 (2)	0.018 (2)	0.017 (2)	−0.0025 (17)	−0.0011 (17)	−0.0080 (18)
N2	0.017 (2)	0.017 (2)	0.034 (3)	−0.0035 (18)	0.001 (2)	−0.008 (2)
O1	0.064 (4)	0.032 (3)	0.097 (5)	−0.031 (3)	−0.062 (3)	0.027 (3)
O2	0.0179 (19)	0.024 (2)	0.031 (2)	−0.0065 (15)	−0.0067 (16)	−0.0058 (17)
O3	0.043 (3)	0.034 (2)	0.063 (3)	−0.006 (2)	−0.001 (2)	−0.028 (2)
O4	0.018 (2)	0.027 (2)	0.054 (3)	−0.0024 (16)	−0.0073 (19)	−0.017 (2)
O5	0.028 (2)	0.028 (2)	0.035 (2)	0.0062 (18)	−0.0103 (18)	−0.0130 (19)
C1	0.027 (3)	0.018 (3)	0.036 (3)	−0.006 (2)	−0.013 (3)	−0.005 (2)
C2	0.020 (3)	0.020 (3)	0.026 (3)	−0.004 (2)	−0.006 (2)	−0.007 (2)
C3	0.024 (3)	0.025 (3)	0.032 (3)	−0.010 (2)	−0.006 (2)	−0.007 (2)
C4	0.029 (3)	0.018 (3)	0.031 (3)	−0.008 (2)	−0.004 (2)	−0.003 (2)
C5	0.023 (3)	0.020 (3)	0.020 (3)	0.000 (2)	−0.001 (2)	−0.007 (2)
C6	0.020 (3)	0.017 (3)	0.023 (3)	−0.004 (2)	−0.003 (2)	−0.005 (2)
C7	0.018 (3)	0.024 (3)	0.026 (3)	−0.005 (2)	−0.005 (2)	−0.011 (2)
C8	0.018 (3)	0.018 (3)	0.026 (3)	−0.002 (2)	−0.012 (2)	−0.006 (2)
C9	0.022 (3)	0.021 (3)	0.025 (3)	−0.001 (2)	−0.015 (2)	−0.005 (2)
C10	0.089 (6)	0.010 (3)	0.022 (3)	0.005 (3)	−0.034 (3)	−0.004 (2)
C11	0.032 (3)	0.042 (4)	0.036 (4)	−0.009 (3)	0.001 (3)	−0.007 (3)
C12	0.033 (3)	0.046 (4)	0.032 (4)	−0.001 (3)	0.000 (3)	−0.011 (3)
C13	0.029 (3)	0.031 (3)	0.032 (3)	0.005 (2)	−0.012 (3)	−0.018 (3)
C14	0.014 (3)	0.021 (3)	0.035 (3)	0.002 (2)	−0.010 (2)	−0.010 (2)
C15	0.016 (3)	0.020 (3)	0.044 (4)	−0.001 (2)	−0.005 (2)	−0.015 (3)
C16	0.014 (3)	0.013 (3)	0.051 (4)	0.000 (2)	−0.013 (3)	−0.005 (3)
C17	0.017 (3)	0.023 (3)	0.029 (3)	−0.002 (2)	−0.011 (2)	−0.005 (2)
C18	0.081 (6)	0.050 (5)	0.069 (6)	0.020 (4)	−0.024 (5)	−0.036 (5)
C19	0.018 (3)	0.020 (3)	0.054 (4)	−0.002 (2)	−0.007 (3)	−0.005 (3)
C20	0.025 (3)	0.021 (3)	0.036 (3)	−0.011 (2)	0.003 (2)	−0.011 (2)
C21	0.034 (3)	0.019 (3)	0.032 (3)	−0.012 (2)	0.003 (3)	−0.006 (2)
C22	0.029 (3)	0.019 (3)	0.022 (3)	0.000 (2)	−0.002 (2)	−0.006 (2)
C23	0.024 (3)	0.019 (3)	0.020 (3)	−0.003 (2)	0.001 (2)	−0.008 (2)
C24	0.017 (3)	0.018 (3)	0.033 (3)	−0.005 (2)	0.001 (2)	−0.009 (2)
C25	0.023 (3)	0.023 (3)	0.025 (3)	0.002 (2)	−0.003 (2)	−0.012 (2)
Cl1	0.0301 (7)	0.0203 (7)	0.0229 (7)	0.0008 (5)	−0.0019 (6)	−0.0076 (5)
O6	0.110 (5)	0.048 (3)	0.025 (3)	0.039 (3)	0.012 (3)	−0.006 (2)
O7	0.058 (4)	0.046 (3)	0.133 (6)	−0.031 (3)	0.002 (4)	−0.034 (4)
O8	0.053 (3)	0.043 (3)	0.032 (3)	0.006 (2)	−0.018 (2)	−0.008 (2)
O9	0.041 (2)	0.018 (2)	0.034 (2)	0.0019 (18)	−0.0040 (19)	−0.0081 (18)
O10	0.0179 (19)	0.0174 (19)	0.028 (2)	−0.0036 (15)	−0.0002 (16)	−0.0041 (16)
O11	0.0197 (19)	0.024 (2)	0.034 (2)	−0.0066 (16)	0.0017 (17)	−0.0143 (18)
O12	0.025 (2)	0.026 (2)	0.098 (4)	−0.0120 (19)	0.014 (2)	−0.027 (3)
O13	0.036 (2)	0.022 (2)	0.031 (2)	0.0036 (18)	−0.0073 (19)	−0.0067 (18)
O14	0.032 (2)	0.022 (2)	0.055 (3)	−0.0050 (18)	−0.020 (2)	−0.010 (2)

### Geometric parameters (Å, °)

**Table:**

Co1—O11	2.086 (4)	C12—C13	1.426 (9)
Co1—O11^i^	2.086 (4)	C12—H12	0.9500
Co1—O10	2.096 (4)	C13—C14	1.422 (9)
Co1—O10^i^	2.096 (4)	C13—H13	0.9500
Co1—O2^i^	2.110 (4)	C14—C15	1.425 (8)
Co1—O2	2.110 (4)	C15—C16	1.380 (9)
N1—C6	1.351 (7)	C16—C17	1.422 (8)
N1—C5	1.350 (7)	C16—C19	1.503 (8)
N1—C7	1.502 (6)	C17—H17	0.9500
N2—C24	1.340 (7)	C18—H18A	0.9800
N2—C20	1.355 (7)	C18—H18B	0.9800
N2—C19	1.516 (7)	C18—H18C	0.9800
O1—C1	1.240 (7)	C19—H19A	0.9900
O2—C1	1.240 (7)	C19—H19B	0.9900
O3—C18	1.370 (9)	C20—C21	1.370 (8)
O3—C15	1.389 (7)	C20—H20	0.9500
O4—C25	1.222 (7)	C21—C22	1.388 (8)
O5—C25	1.217 (7)	C21—H21	0.9500
C1—C2	1.517 (8)	C22—C23	1.389 (8)
C2—C6	1.379 (7)	C22—H22	0.9500
C2—C3	1.386 (8)	C23—C24	1.384 (8)
C3—C4	1.383 (8)	C23—C25	1.544 (8)
C3—H3	0.9500	C24—H24	0.9500
C4—C5	1.378 (8)	Cl1—O8	1.410 (5)
C4—H4	0.9500	Cl1—O7	1.413 (5)
C5—H5	0.9500	Cl1—O6	1.423 (5)
C6—H6	0.9500	Cl1—O9	1.446 (4)
C7—C8	1.509 (7)	O10—H10A	0.84 (4)
C7—H7A	0.9900	O10—H10B	0.85 (4)
C7—H7B	0.9900	O11—H11A	0.87 (4)
C8—C17	1.382 (8)	O11—H11B	0.86 (4)
C8—C9	1.424 (8)	O12—H12A	0.83 (4)
C9—C10	1.419 (9)	O12—H12B	0.87 (4)
C9—C14	1.421 (8)	O13—H13A	0.86 (4)
C10—C11	1.425 (10)	O13—H13B	0.86 (4)
C10—H10	0.9500	O14—H14A	0.86 (4)
C11—C12	1.402 (10)	O14—H14B	0.86 (4)
C11—H11	0.9500		
			
O11—Co1—O11^i^	180.0	C13—C12—H12	120.5
O11—Co1—O10	89.89 (15)	C14—C13—C12	120.8 (5)
O11^i^—Co1—O10	90.11 (15)	C14—C13—H13	119.6
O11—Co1—O10^i^	90.11 (15)	C12—C13—H13	119.6
O11^i^—Co1—O10^i^	89.89 (15)	C9—C14—C13	120.4 (5)
O10—Co1—O10^i^	179.999 (1)	C9—C14—C15	119.7 (5)
O11—Co1—O2^i^	90.63 (15)	C13—C14—C15	119.8 (5)
O11^i^—Co1—O2^i^	89.37 (15)	C16—C15—O3	123.0 (5)
O10—Co1—O2^i^	91.78 (15)	C16—C15—C14	120.4 (5)
O10^i^—Co1—O2^i^	88.22 (15)	O3—C15—C14	116.3 (6)
O11—Co1—O2	89.37 (15)	C15—C16—C17	119.5 (5)
O11^i^—Co1—O2	90.63 (15)	C15—C16—C19	120.9 (5)
O10—Co1—O2	88.22 (15)	C17—C16—C19	119.1 (6)
O10^i^—Co1—O2	91.78 (15)	C8—C17—C16	121.6 (6)
O2^i^—Co1—O2	179.998 (1)	C8—C17—H17	119.2
C6—N1—C5	121.2 (4)	C16—C17—H17	119.2
C6—N1—C7	119.1 (4)	O3—C18—H18A	109.5
C5—N1—C7	119.6 (4)	O3—C18—H18B	109.5
C24—N2—C20	120.7 (5)	H18A—C18—H18B	109.5
C24—N2—C19	122.8 (4)	O3—C18—H18C	109.5
C20—N2—C19	116.5 (4)	H18A—C18—H18C	109.5
C1—O2—Co1	127.1 (4)	H18B—C18—H18C	109.5
C18—O3—C15	117.9 (6)	C16—C19—N2	115.7 (4)
O2—C1—O1	126.4 (5)	C16—C19—H19A	108.3
O2—C1—C2	116.4 (5)	N2—C19—H19A	108.3
O1—C1—C2	117.2 (5)	C16—C19—H19B	108.3
C6—C2—C3	118.9 (5)	N2—C19—H19B	108.3
C6—C2—C1	120.1 (5)	H19A—C19—H19B	107.4
C3—C2—C1	121.0 (5)	N2—C20—C21	120.3 (5)
C4—C3—C2	119.6 (5)	N2—C20—H20	119.8
C4—C3—H3	120.2	C21—C20—H20	119.8
C2—C3—H3	120.2	C20—C21—C22	119.6 (5)
C5—C4—C3	119.8 (5)	C20—C21—H21	120.2
C5—C4—H4	120.1	C22—C21—H21	120.2
C3—C4—H4	120.1	C21—C22—C23	119.7 (5)
N1—C5—C4	119.8 (5)	C21—C22—H22	120.1
N1—C5—H5	120.1	C23—C22—H22	120.1
C4—C5—H5	120.1	C24—C23—C22	118.2 (5)
N1—C6—C2	120.6 (5)	C24—C23—C25	119.7 (5)
N1—C6—H6	119.7	C22—C23—C25	122.1 (5)
C2—C6—H6	119.7	N2—C24—C23	121.4 (5)
N1—C7—C8	114.6 (4)	N2—C24—H24	119.3
N1—C7—H7A	108.6	C23—C24—H24	119.3
C8—C7—H7A	108.6	O5—C25—O4	129.2 (5)
N1—C7—H7B	108.6	O5—C25—C23	115.8 (5)
C8—C7—H7B	108.6	O4—C25—C23	115.0 (5)
H7A—C7—H7B	107.6	O8—Cl1—O7	109.3 (4)
C17—C8—C9	119.5 (5)	O8—Cl1—O6	110.4 (3)
C17—C8—C7	118.3 (5)	O7—Cl1—O6	109.7 (4)
C9—C8—C7	121.7 (5)	O8—Cl1—O9	109.5 (3)
C10—C9—C14	117.9 (5)	O7—Cl1—O9	109.0 (3)
C10—C9—C8	122.8 (5)	O6—Cl1—O9	108.9 (3)
C14—C9—C8	119.3 (5)	Co1—O10—H10A	116 (5)
C9—C10—C11	121.7 (5)	Co1—O10—H10B	96 (4)
C9—C10—H10	119.1	H10A—O10—H10B	109 (4)
C11—C10—H10	119.1	Co1—O11—H11A	116 (4)
C12—C11—C10	120.0 (6)	Co1—O11—H11B	124 (4)
C12—C11—H11	120.0	H11A—O11—H11B	105 (4)
C10—C11—H11	120.0	H12A—O12—H12B	107 (5)
C11—C12—C13	119.0 (6)	H13A—O13—H13B	105 (4)
C11—C12—H12	120.5	H14A—O14—H14B	105 (4)
			
O11—Co1—O2—C1	−107.3 (5)	C10—C9—C14—C15	−178.3 (5)
O11^i^—Co1—O2—C1	72.7 (5)	C8—C9—C14—C15	−0.2 (7)
O10—Co1—O2—C1	162.8 (5)	C12—C13—C14—C9	3.4 (8)
O10^i^—Co1—O2—C1	−17.2 (5)	C12—C13—C14—C15	−178.5 (5)
O2^i^—Co1—O2—C1	40 (29)	C18—O3—C15—C16	−84.6 (8)
Co1—O2—C1—O1	−4.0 (10)	C18—O3—C15—C14	101.7 (8)
Co1—O2—C1—C2	174.9 (4)	C9—C14—C15—C16	−0.8 (8)
O2—C1—C2—C6	173.3 (5)	C13—C14—C15—C16	−178.9 (5)
O1—C1—C2—C6	−7.7 (9)	C9—C14—C15—O3	173.1 (5)
O2—C1—C2—C3	−8.1 (9)	C13—C14—C15—O3	−5.0 (7)
O1—C1—C2—C3	171.0 (7)	O3—C15—C16—C17	−172.9 (5)
C6—C2—C3—C4	1.2 (9)	C14—C15—C16—C17	0.6 (8)
C1—C2—C3—C4	−177.5 (6)	O3—C15—C16—C19	−1.3 (8)
C2—C3—C4—C5	−0.4 (9)	C14—C15—C16—C19	172.2 (5)
C6—N1—C5—C4	1.7 (8)	C9—C8—C17—C16	−1.7 (8)
C7—N1—C5—C4	178.1 (5)	C7—C8—C17—C16	170.2 (5)
C3—C4—C5—N1	−1.1 (9)	C15—C16—C17—C8	0.7 (8)
C5—N1—C6—C2	−1.0 (8)	C19—C16—C17—C8	−171.1 (5)
C7—N1—C6—C2	−177.4 (5)	C15—C16—C19—N2	92.2 (7)
C3—C2—C6—N1	−0.5 (8)	C17—C16—C19—N2	−96.1 (6)
C1—C2—C6—N1	178.2 (5)	C24—N2—C19—C16	16.4 (8)
C6—N1—C7—C8	−61.4 (6)	C20—N2—C19—C16	−166.5 (5)
C5—N1—C7—C8	122.1 (5)	C24—N2—C20—C21	−2.2 (8)
N1—C7—C8—C17	114.7 (5)	C19—N2—C20—C21	−179.3 (5)
N1—C7—C8—C9	−73.6 (6)	N2—C20—C21—C22	−0.5 (9)
C17—C8—C9—C10	179.5 (5)	C20—C21—C22—C23	2.6 (9)
C7—C8—C9—C10	7.8 (8)	C21—C22—C23—C24	−2.2 (8)
C17—C8—C9—C14	1.5 (7)	C21—C22—C23—C25	178.1 (5)
C7—C8—C9—C14	−170.2 (5)	C20—N2—C24—C23	2.6 (8)
C14—C9—C10—C11	−4.3 (9)	C19—N2—C24—C23	179.6 (5)
C8—C9—C10—C11	177.6 (6)	C22—C23—C24—N2	−0.4 (8)
C9—C10—C11—C12	5.8 (10)	C25—C23—C24—N2	179.3 (5)
C10—C11—C12—C13	−2.5 (9)	C24—C23—C25—O5	−166.5 (5)
C11—C12—C13—C14	−2.0 (9)	C22—C23—C25—O5	13.3 (8)
C10—C9—C14—C13	−0.2 (8)	C24—C23—C25—O4	14.9 (8)
C8—C9—C14—C13	177.9 (5)	C22—C23—C25—O4	−165.4 (5)

Symmetry codes: (i) −*x*+3, −*y*+1, −*z*+1.

### Hydrogen-bond geometry (Å, °)

**Table:**

*D*—H···*A*	*D*—H	H···*A*	*D*···*A*	*D*—H···*A*
O10—H10A···O13^ii^	0.84 (4)	1.83 (4)	2.665 (5)	171 (6)
O10—H10B···O1^i^	0.85 (4)	1.84 (4)	2.668 (6)	166 (6)
O11—H11A···O12^ii^	0.87 (4)	1.83 (4)	2.690 (6)	174 (7)
O11—H11B···O14^iii^	0.86 (4)	1.85 (4)	2.701 (5)	173 (6)
O12—H12A···O1	0.83 (4)	1.84 (5)	2.654 (6)	164 (8)
O12—H12B···O4	0.87 (4)	1.91 (4)	2.772 (6)	173 (8)
O13—H13A···O5^iv^	0.86 (4)	1.94 (4)	2.787 (6)	172 (7)
O13—H13B···O9^v^	0.86 (4)	1.95 (4)	2.815 (6)	175 (7)
O13—H13B···Cl1^v^	0.86 (4)	2.69 (6)	3.451 (4)	147 (6)
O14—H14A···O4^ii^	0.86 (4)	1.88 (4)	2.724 (6)	165 (7)
O14—H14B···O5^vi^	0.86 (4)	1.98 (4)	2.811 (6)	160 (7)

Symmetry codes: (ii) −*x*+2, −*y*+1, −*z*+1; (i) −*x*+3, −*y*+1, −*z*+1; (iii) *x*+1, *y*, *z*; (iv) −*x*+2, −*y*, −*z*+1; (v) −*x*+1, −*y*, −*z*+1; (vi) *x*−1, *y*+1, *z*.
